# Moderators and predictors of treatment outcome following adjunctive internet‐delivered emotion regulation therapy relative to treatment as usual alone for adolescents with nonsuicidal self‐injury disorder: Randomized controlled trial

**DOI:** 10.1002/jcv2.12243

**Published:** 2024-05-06

**Authors:** Olivia Ojala, Hugo Hesser, Kim L. Gratz, Matthew T. Tull, Erik Hedman‐Lagerlöf, Hanna Sahlin, Brjánn Ljótsson, Clara Hellner, Johan Bjureberg

**Affiliations:** ^1^ Centre for Psychiatry Research Department of Clinical Neuroscience Karolinska Institutet, & Stockholm Health Care Services Stockholm Sweden; ^2^ School of Law, Psychology and Social Work Örebro University Örebro Sweden; ^3^ Department of Psychology University of Toledo Toledo Ohio USA; ^4^ Division of Psychology Department of Clinical Neuroscience Karolinska Institutet Stockholm Sweden

**Keywords:** emotion regulation, internet‐delivered therapy, moderator, nonsuicidal self‐injury, self‐injurious behaviors

## Abstract

**Background:**

Despite the wide‐ranging negative consequences of nonsuicidal self‐injury (NSSI), there are few evidence‐based treatments for NSSI among adolescents and little is known about what treatments that work best for whom. The objective of this study was to investigate moderators (i.e., for whom a specific treatment works) and predictors (i.e., factors associated with treatment outcome independent of treatment type) of treatment outcome in a randomized clinical trial comparing internet‐delivered emotion regulation individual therapy for adolescents (IERITA) plus treatment as usual (TAU) to TAU alone.

**Methods:**

Adolescents (*N* = 166; mean [SD] age = 15.0 [1.2] years) with NSSI disorder were randomized to IERITA plus TAU (*n* = 84) or TAU‐only (*n* = 82). Adolescent emotion regulation difficulties, suicidality, NSSI frequency, depressive symptoms, sleep difficulties, global functioning, and age, and parental invalidation, were measured pre‐treatment and investigated as moderators and predictors of treatment outcome (i.e., NSSI frequency during treatment and for 4 weeks post‐treatment). A zero‐inflated negative binomial generalized linear mixed effects regression model was used to estimate the rate of NSSI change as a function of both treatment condition and moderator/predictor.

**Results:**

No significant moderators of treatment outcome were found. Parental invalidation was a significant predictor of treatment outcome regardless of treatment condition, such that high levels of parental invalidation pre‐treatment were associated with a less favorable NSSI frequency.

**Conclusions:**

We did not find evidence of a differential treatment effect as a function of any of the examined client factors. Future research should investigate moderation in larger samples and with sufficient statistical power to detect moderation effects of smaller magnitude. Results suggest that parental invalidation may have a negative impact on treatment response and highlight the importance of further investigating parental invalidation in the context of NSSI treatments.

## Graphical abstract

Internet‐delivered emotion regulation individual therapy for adolescents (IERITA) delivered adjunctive to treatment as usual could potentially be applicable to a wide range of adolescents with nonsuicidal self‐injury disorder. Parental invalidation was associated with poorer treatment outcome regardless of treatment condition.

**Key points**

- Internet‐delivered emotion regulation individual therapy for adolescents (IERITA) delivered adjunctive to treatment as usual (TAU) is superior in treating adolescent nonsuicidal self‐injury (NSSI) compared to TAU only.
- Emotion regulation difficulties, parental invalidation, suicidality, prior NSSI, depressive symptoms, sleep difficulties, global functioning, and age did not moderate treatment outcome.
- Parental invalidation was associated with poorer treatment outcome regardless of treatment condition.
- IERITA could potentially be applicable to a wide range of adolescents with NSSI disorder.
- Parental invalidation may have a negative impact on treatment response and deserves further investigation in the context of NSSI treatments.

## BACKGROUND

Clinicians strive to offer the most effective treatment based on the unique needs of any given individual. This requires both the availability of evidence‐based treatments for a client's particular difficulties and, ideally, information on for whom the treatment is most effective. Currently, there is a relative lack of brief, transportable treatments for adolescents engaging in nonsuicidal self‐injury (NSSI; Glenn et al., 2019). Given the elevated risk of several adverse outcomes associated with adolescent NSSI (Bjureberg et al., 2019; Mars et al., 2014; Ohlis et al., 2020; Wilkinson et al., 2018), including suicide attempts (Ribeiro et al., 2016), such treatments are greatly needed. Internet‐delivered treatment is one way of increasing accessibility to evidence‐based psychological treatments.

Our group recently published the first randomized clinical trial (RCT) of an internet‐delivered treatment for adolescents with NSSI disorder (NSSID; American Psychiatric Association, 2013). Results of this trial provided support for the efficacy of internet‐delivered emotion regulation individual therapy for adolescents (IERITA) delivered adjunctive to treatment as usual (TAU), relative to TAU only, in the reduction of NSSI (Bjureberg et al., 2023). Although IERITA was designed to be helpful for a broad range of adolescents with NSSI, it is possible that the efficacy of this treatment may vary for different clients, as differences in treatment effect is usually expected (Kraemer et al., 2006). Understanding who may be more or less likely to benefit from IERITA is important for clinical decision making and optimizing individual treatment outcome. Further, investigating moderators (i.e., for whom a specific treatment works; Baron & Kenny, 1986; Kraemer et al., 2002) and predictors (i.e., factors associated with treatment outcome independent of treatment type; Kraemer et al., 2002) could inform treatment development and refinement and guide further research with respect to design questions (e.g., stratification and guidance for power calculations). Finally, increased knowledge of moderators and predictors of treatment outcome following evidence‐based treatments for adolescent NSSI can also advance our understanding of the treatment of adolescent self‐injury in general (Glenn et al., 2019). To date, there are only a few studies of moderators or predictors of treatment outcome for treatments targeting and evaluating NSSI among adolescents (Adrian et al., 2019; Edinger et al., 2020) and adults (Andover et al., 2020; Gratz et al., 2014; Sahlin et al., 2019), with none examining these for internet‐delivered treatments specifically.

As a secondary analysis of the RCT on IERITA (Bjureberg et al., 2023), we aimed to study moderators and predictors of treatment outcome to this adjunctive treatment (relative to TAU only). In accordance with recommendation (Kraemer et al., 2002), eight specific client factors were selected a priori, based on theoretical and empirical grounds.

The first selected factor of interest was emotion regulation difficulties. Results from the RCT of IERITA suggest that the reduction in NSSI is explained, at least in part, by decreases in emotion regulation difficulties (which is the theorized treatment mechanism; Bjureberg et al., 2023). In a previous study (Adrian et al., 2019), adolescents with greater emotion regulation difficulties had better treatment effect of a treatment that targets emotion regulation difficulties (i.e., dialectical behavior therapy for adolescents [DBT‐A]) compared to a treatment with another focus (i.e., individual and group supportive therapy [IGST]). In addition, greater emotion regulation difficulties were found to predict better treatment response (i.e., less borderline personality disorder [BPD] symptoms) to a brief emotion regulation group therapy (ERGT; Gratz & Gunderson, 2006); the treatment that IERITA was developed from, among adult women with BPD and NSSI (Gratz et al., 2014). However, emotion regulation difficulties did not predict change in the outcome of NSSI during ERGT (Gratz et al., 2014; Sahlin et al., 2019).

Both IERITA and ERGT draw from dialectical behavior therapy (DBT; Linehan, 1993), which posits that invalidating environments contribute to the etiology and maintenance of emotion regulation difficulties and self‐injury. In IERITA, parental invalidation (judging, rejecting, or devaluing their adolescents' emotions) is targeted in the parent program. For adolescents receiving either DBT‐A or IGST, high pre‐treatment parental invalidation predicted greater self‐injury frequency at post‐treatment (Adrian et al., 2018). However, how parental invalidation affects the treatment outcome for a specific adolescent NSSI treatment (relative to another treatment) is unknown.

We also aimed to examine suicidality as a potential moderator or predictor. The extent to which client suicidality influences treatment outcome to NSSI treatments is an important clinical question, particularly for online treatments, which may raise greater concerns about the management of suicidality (Gilmore & Ward‐Ciesielski, 2019). To date, it is unclear whether level of suicidality influence treatment outcome for adolescent NSSI. For adults receiving ERGT, the presence of a suicide attempt within the 3 months before starting ERGT predicted better treatment outcome (Sahlin et al., 2019); however, lifetime or past year suicide attempts did not influence treatment outcome (Gratz et al., 2014; Sahlin et al., 2019).

Severity of NSSI is another important factor to consider, particularly when considering the potential utility of a brief internet‐delivered intervention for adolescents with highly frequent NSSI. For adults with NSSI, high pre‐treatment NSSI frequency has been shown to both predict and moderate better treatment outcome (Andover et al., 2020; Gratz et al., 2014; Sahlin et al., 2019). It is possible that the impairment and distress associated with more frequent NSSI (Muehlenkamp & Brausch, 2016; Muehlenkamp et al., 2017) increases treatment motivation and engagement in treatments focused on NSSI. Notably, however, for adolescents receiving DBT‐A or IGST, greater pre‐treatment NSSI frequency predicted poorer treatment response independent of treatment condition (Adrian et al., 2019).

There is evidence that both depressive symptoms and sleep difficulties co‐occur with NSSI in adolescents (Sun et al., 2023) and may maintain NSSI over time (Latina et al., 2021; Marshall et al., 2013). Current research examining these factors as moderators or predictors of treatment outcome considering NSSI among adolescents is limited. For adolescents receiving DBT‐A, IGST or enhanced usual care, higher levels of depression pre‐treatment predicted poorer treatment response regarding self‐injury with or without suicide intent (Berk et al., 2022; Dibaj et al., 2023). For women with recurrent NSSI receiving ERGT, having a depressive disorder predicted better treatment response in one study (Sahlin et al., 2019), but not in another (Gratz et al., 2014). Additionally, greater pre‐treatment sleep difficulties among adolescents receiving a DBT‐informed family treatment, predicted greater suicidal behavior after treatment (Babeva et al., 2020).

Finally, given that some adolescents who have received IERITA reported difficulties with the responsibility and organization required by the treatment (Simonsson et al., 2021), IERITA could potentially be more challenging for adolescents with lower levels of global functioning or younger age. Although no research to date has examined global functioning as a predictor or moderator of treatment response to NSSI treatments among adolescents, age has been investigated but not found to predict or moderate treatment outcome for adolescents receiving DBT‐A or IGST (Adrian et al., 2019).

In sum, emotion regulation difficulties, parental invalidation, suicidality, NSSI frequency, depressive symptoms, sleep difficulties, global functioning, and age are theoretically relevant to investigate as putative moderators/predictors of treatment response to IERITA. Given the relative lack of research in this area, as well as mixed findings of the studies that have been conducted, we considered the current investigation exploratory.

## METHODS

### Study design

In this 3‐site RCT (Bjureberg et al., 2023), participating families (*N* = 166; including an adolescent and a parent/legal guardian) were randomized to either IERITA delivered adjunctive to TAU (*n* = 84), or TAU only (*n* = 82). Data was collected between November 2017 and January 2021.

### Participants

The inclusion criteria for adolescents in the trial (Bjureberg et al., 2023) were: (a) 13–17 years; (b) fulfilling diagnostic criteria for NSSID; (c) having engaged in ≥1 NSSI episode during the past month; and (d) having at least one guardian (hereafter referred to as parent) who could participate in the parent program. Exclusion criteria for the adolescents in the trial (Bjureberg et al., 2023) were: (a) immediate suicide risk; (b) diagnosis of psychotic or bipolar I disorder or current (past month) substance dependence; (c) other primary psychiatric disorder requiring different and immediate treatment (e.g., severe anorexia nervosa); (d) insufficient understanding of the Swedish language; (e) life circumstances that could interfere with or prevent treatment participation, or that required immediate intervention (e.g., violence in close relationships); and (f) a clinician assessed global functioning level of Children's Global Assessment Scale (CGAS; Shaffer et al., 1983) of <40.

Many of the participants were clinician‐referred (*n* = 101, 61%). Most participants were female (*n* = 154, 93%), with a mean age of 15.0 (SD = 1.2). The average number of co‐occurring disorders was 1.9 (SD = 1.6), and the most common co‐occurring disorders were major depressive disorder (*n* = 97, 58%) and social anxiety disorder (*n* = 47, 28%). Many participants had ongoing therapy at inclusion (*n* = 116, 70%), with supportive therapy (*n* = 49, 42%) and cognitive behavioral therapy (*n* = 38, 33%) being the most common types. Approximately one‐third of the sample had ongoing psychopharmacological treatment at inclusion (*n* = 56, 34%), with antidepressants being the most common type (*n* = 35, 21%). More detailed participant information is presented in the original report (Bjureberg et al., 2023).

### Procedure

Participating families went through a brief telephone screening and a following face‐to‐face assessment. All assessors were clinical psychologists or psychotherapists. Families were then randomized to treatment conditions in blocks of four or six per treatment site. Parents and adolescents completed self‐report measures pre‐treatment, during treatment, and post‐treatment. The CONSORT flow diagram for the trial is presented in Figure S1.

### Intervention

IERITA is a 12‐week acceptance‐based emotion regulation behavioral therapy developed from the face‐to‐face manuals of emotion regulation individual therapy for adolescents (Bjureberg et al., 2017), ERGT (Gratz & Gunderson, 2006) and DBT‐A (Miller et al., 2007), with the goal of decreasing NSSI by targeting emotion regulation difficulties. Across 11 weekly modules, adolescents receive interventions targeting emotional awareness and acceptance, impulse control, self‐validation, identifying and engaging in valued directions, and non‐avoidant emotion regulation skills. In addition, a supplementary mobile app could be used to facilitate the daily implementation of skills. IERITA also includes a separate parent program in six modules. The parent program includes psychoeducation about NSSI, emotional awareness, validation, behavioral activation, and response prevention. Both adolescent and parent had separate asynchronous contact with a therapist (psychologist/psychotherapist) in the secure online platform.

In this study, TAU delivered in both conditions consisted of regular care delivered by clinicians uninvolved in the study based on the individual's needs (i.e., co‐occurring disorder[s], severity level, and global functioning). This meant that participants in IERITA plus TAU usually had contact with one IERITA therapist and one community clinician. All families were recommended and/or referred to TAU before randomization. Adolescents also completed online questionnaires on a weekly basis throughout the intervention period. If deterioration in mental health was detected, the study therapist would contact the family and advise them to seek support. A more detailed description of the interventions and safety routines is found elsewhere (Bjureberg et al., 2023).

### Measures

#### Outcome

NSSI frequency was self‐reported using the youth version of the deliberate self‐harm inventory (DSHI‐Y; Gratz et al., 2012). The DSHI has shown adequate construct, convergent, and discriminant validity (Gratz, 2001). In this study, the DSHI‐Y was used to assess past‐week presence and frequency of six common NSSI behaviors. The DSHI‐Y was administered pre‐treatment (week 0, before randomization), during treatment (week 1–12), and 4 weeks post‐treatment (primary endpoint).

### Potential moderators/predictors

#### Emotion regulation difficulties

Emotion regulation difficulties were self‐reported pre‐treatment (i.e., week 0) with the difficulties in emotion regulation scale (DERS; Gratz & Roemer, 2004). The DERS consists of 36 items rated on a 5‐point scale, resulting in a score between 36 and 180. Higher scores indicate greater emotion regulation difficulties. The DERS has shown satisfactory to excellent internal consistency and construct validity among adolescents (Neumann et al., 2010; Weinberg & Klonsky, 2009). Internal consistency in this sample was excellent (*α* = .90).

#### Parental invalidation

Parental invalidation was self‐reported pre‐treatment by the parent using the Coping with Children's Negative Emotions Scale (CCNES)—Adolescent version (Fabes et al., 1990). The CCNES—Adolescent version presents parents with nine hypothetical scenarios involving adolescent distress and asks parents to rate how likely it is that they would respond in various ways. In this study, the likelihood of using the response style “minimization” (i.e., discounting or devaluing the child's negative emotions/emotional expressions) was used to measure parental invalidation. Parents rate the likelihood of minimization on a 7‐point scale for each scenario. Scores are then summed and divided by the number of scenarios (i.e., nine), with higher scores indicating greater levels of parental invalidation. The CCNES has shown acceptable internal consistency, test‐retest reliability, and construct validity (Fabes et al., 2002). Internal consistency for the minimization subscale in this sample was acceptable (*α* = .79).

#### Suicidality

Suicidality was clinician‐assessed at the pre‐treatment face‐to‐face interview and measured with the Mini International Neuropsychiatric Interview for Children and Adolescents (MINI‐KID), version 6 (Sheehan et al., 1998). Suicidality was rated on a scale from 0 to 87 with identified anchors for low (1–8), moderate (9–16), and high (≥17) suicidality (Sheehan et al., 2010).The presence versus abscence of high suicidality as a binary variable was used in this study. The MINI‐KID has been shown to have acceptable to excellent test‐retest and interrater reliability and acceptable to excellent concurrent validity (Högberg et al., 2019; Sheehan et al., 2010).

#### Prior NSSI frequency

Past month NSSI frequency was clinician‐assessed at the pre‐treatment face‐to‐face interview and measured with the DSHI‐Y (Gratz et al., 2012), which assessed the presence and frequency of NSSI during the last 30 days.

#### Depressive symptoms

Depressive symptoms were self‐reported pre‐treatment using the depression subscale of the 21‐item depression anxiety and stress scales (DASS‐21; Antony et al., 1998; Lovibond & Lovibond, 1995). Depressive symptoms are rated across seven items on a 4‐point scale resulting in a score between 0 and 21. Higher scores indicate greater depressive symptoms. The DASS‐21 has shown good internal consistency and convergent validity among adolescents (Evans et al., 2021; Tully et al., 2009). Internal consistency in this sample was excellent (*α* = .91).

#### Sleep difficulties

Sleep difficulties were self‐reported pre‐treatment and measured with the Insomnia Severity Index (ISI; Bastien et al., 2001). The ISI consists of seven items rated on a 5‐point scale, resulting in a score between 0 and 28. Higher scores indicate greater sleep difficulties. The ISI has shown acceptable internal consistency and construct validity among adolescents (Azita et al., 2021; Gerber et al., 2016). Internal consistency in this sample was good (*α* = .80).

#### Global functioning

Global functioning was clinician‐assessed at the pre‐treatment face‐to‐face interview and measured with the children's global assessment scale (CGAS; Shaffer et al., 1983). This measure produces a score from 0 to 100, with higher scores indicating greater global functioning. The CGAS has shown concurrent and discriminative validity and moderate to excellent interrater reliability (Bird et al., 1987; Lundh et al., 2010; Shaffer et al., 1983).

#### Age

Adolescent age was reported by caregivers at the pre‐treatment face‐to‐face interview.

### Statistical analysis

Moderated treatment effects were evaluated according to the intention‐to‐treat principle and under the missing at random assumption. The analysis included the weekly reports of NSSI frequency (DSHI‐Y) measured pre‐treatment, once every week during treatment, and once a week for 4 weeks post‐treatment (primary endpoint). A zero‐inflated negative binomial generalized linear mixed effects regression model was fitted to the NSSI counts to estimate the rate of change as a function of both treatment condition and moderator variable. The model included fixed effects of time, treatment condition, moderator, and their interactions, and subject‐specific random effects for intercept and linear time for the count part of the model. Continuous variables were grand mean centered before being entered in the model as predictors. The key parameters in the model evaluated whether pre‐treatment levels of the variables tested as a predictor or moderator influenced change over time across treatment conditions (i.e., two‐way interaction between predictor and time) and differential rate of change as a function of treatment condition (i.e., three‐way interaction between moderator, treatment condition, and time). If an interaction was statistically significant, we probed it by examining growth trajectories at sample average, high, and low pre‐treatment levels of the moderator (+/−1 SD). Correlation analysis between variables tested as moderators or predictors were conducted before conducting the primary analyses. The correlations between moderators were deemed appropriate (i.e., *r* < .7 as has been suggested an appropriate indicator [Dormann et al., 2013], see Table S1); hence, all moderators were deemed relevant to test separately. A separate analysis was evaluated for each moderator. A 5% two‐tailed test of significance was used throughout without correction for multiple testing, consistent with the hypothesis‐generating approach of testing moderators in RCTs (e.g., Kraemer et al., 2002). Rather than examining age as a continuous variable, a sensitivity analysis was conducted by dividing the sample into younger (i.e., 13–14 years [*n* = 81]) versus older (i.e., 15–17 years [*n* = 85]) age groups.

The analyses were conducted using the statistical software R version 4.1.2 (R Core Team, 2021). The package GLMMadaptive (Rizopoulos, 2021) was used for fitting generalized linear mixed effects regression models and estimating marginal coefficients (Hedeker et al., 2018) with robust standard errors (i.e., Sandwich estimator).

## RESULTS

### Descriptive information

Descriptive data on moderators/predictors are presented in Tables 1 and S2; descriptives on outcome measures are presented in Table S3.

**TABLE 1:** Descriptive results of the outcome and moderators/predictors.

Moderator/predictor	IERITA + TAU (*n* = 84)	TAU‐only (*n* = 82)
	Mean (SD)	Mean (SD)
Age	15.0 (1.3)	15.0 (1.2)
Depressive symptoms	11.9 (5.0)	12.1 (4.6)
ER difficulties	128.4 (20.3)	130.2 (19.4)
Global functioning	54.3 (5.6)	54.8 (6.8)
Parental invalidation	2.9 (1.0)	2.6 (1.0)
Sleep difficulties	10.8 (5.4)	11.3 (6.0)
Prior past‐month NSSI	16.0 (22.2)	12.9 (13.8)

	*n* (%)	*n* (%)
High suicidality	26 (31.0)	24 (29.3)

*Note*: All measures of moderator/predictor variables had complete data.

Abbreviations: ER, emotion regulation; IERITA, internet‐delivered emotion regulation individual therapy; NSSI, nonsuicidal self‐injury; Q, quantile; SD, standard deviation; TAU, treatment as usual.

### Moderators and predictors

**FIGURE 1:**
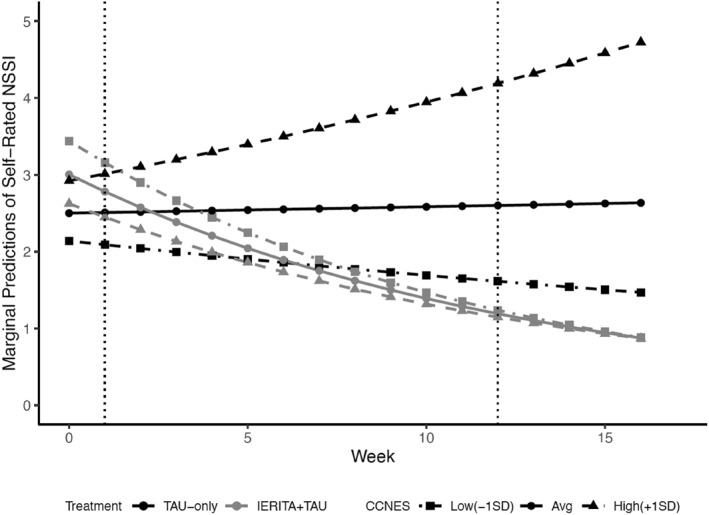
Marginal predictions of self‐rated NSSI episodes at pre‐treatment (week 0), every week during treatment (week 1 to 12), and 4 weeks post‐treatment as a function of treatment condition and pre‐treatment levels of parental invalidation. CCNES, coping with children's negative emotions scale; IERITA, internet‐delivered emotion regulation individual therapy for adolescents; NSSI, nonsuicidal self‐injury; TAU, treatment as usual.

Table 2 presents the key results of the fitted generalized linear mixed effects regression models evaluating moderated treatment effects on self‐reported NSSI episodes (DSHI‐Y). Detailed results from the fitted generalized linear mixed effects regression models (e.g., intercept, main effects) are presented in Table S4. In line with the findings from the primary outcome analysis presented in the original report (Bjureberg et al., 2023), the interaction between treatment and time was statistically significant, with a higher rate of change (reduction) of self‐rated NSSI episodes in the ERITA plus TAU group relative to the TAU‐only (at the sample average level of the moderator; Table S4). However, none of the key interaction terms that included the moderator/predictor variable were statistically significant, with one exception: that is, results revealed a positive interaction between time and parental invalidation (CCNES; see Table 2), indicating that parental invalidation measured pre‐treatment is a significant predictor of treatment response. As illustrated in Figure 1, higher pre‐treatment levels of parental invalidation (+1 SD) were associated with fewer improvements in NSSI frequency in the ERITA plus TAU condition, and an increase in NSSI frequency in the TAU‐only condition. Thus, higher reported pre‐treatment levels of parental invalidation were associated with less favorable outcomes across both conditions. None of the three‐way interactions that tested for moderated effects as a function of treatment condition was statistically significant. Results from the sensitivity analysis examining age groups showed non‐significant prediction or moderation effects (Table S5), in line with the main findings.

**TABLE 2:** Key parameter estimates obtained from generalized linear mixed models examining change in self‐rated NSSI episodes from pre‐treatment to post‐treatment as a function of treatment condition.

	Estimate (*b*)	SE	*z*‐Value	*p*‐Value
Age				
Moderator	−0.018	0.017	−1.064	.287
Predictor	0.018	0.011	1.666	.096
High suicidality				
Moderator	0.015	0.046	0.329	.742
Predictor	−0.004	0.029	−0.145	.885
Global functioning				
Moderator	−0.004	0.004	−0.974	.330
Predictor	−0.001	0.001	−0.712	.477
Parental invalidation				
Moderator	−0.019	0.017	−1.068	.285
Predictor	0.027	0.011	2.455	.014
Depressive symptoms				
Moderator	0.005	0.005	1.181	.238
Predictor	−0.001	0.003	−0.246	.806
ER difficulties				
Moderator	0.002	0.001	1.600	.110
Predictor	0.001	0.001	1.256	.209
Sleep problems				
Moderator	−0.003	0.004	−0.713	.476
Predictor	0.001	0.003	0.430	.667
Prior past‐month NSSI				
Moderator	0.036	0.042	0.839	.401
Predictor	−0.021	0.027	−0.785	.433

*Note*: Moderator corresponds to the interactions of client factor × time × treatment. Predictor corresponds to the interaction of client factor × time. All parameters are presented in Table S2. Marginal coefficients with robust standard errors are presented. Reference group for treatment is treatment as usual (control condition). Reference group for high suicidality is moderate or low suicidality. Reference group for prior NSSI is <8 episodes.

Abbreviations: ER, emotion regulation; NSSI, nonsuicidal self‐injury; SE, standard error.

## DISCUSSION

The purpose of this study was to examine several theoretically and empirically derived moderators and predictors of treatment outcome in an RCT comparing IERITA plus TAU to TAU only among adolescents with NSSID. The results revealed no significant moderating effects for any of the eight variables tested. Only parental invalidation was identified as a nonspecific predictor of treatment response, with higher pre‐treatment parental invalidation associated with a less favorable outcome in both conditions.

Several of the variables tested in this study have been examined in previous studies of moderators and predictors of treatment outcome for treatments targeting and evaluating NSSI, with mixed results. Neither age nor prior NSSI frequency emerged as significant moderators of treatment outcome among adolescents in a previous RCT of a similar sample size (*N* = 173) comparing DBT‐A to IGST (Adrian et al., 2019). Likewise, in a small RCT (*N* = 33) comparing a brief behavioral treatment to TAU among adults with NSSI, age, suicidality, depressive symptoms, and emotion regulation difficulties did not emerge as moderators of treatment outcome (Andover et al., 2020). However, both pre‐treatment emotion regulation difficulties (Adrian et al., 2019) and past NSSI frequency (Andover et al., 2020) have been found to moderate treatment response to NSSI treatments in previous research. The non‐significant moderation effects found in this study could have several potential explanations. First, the absence of any significant moderators of treatment outcome could suggest that IERITA has utility across a wide range of adolescents with NSSID with respect to pre‐treatment emotion regulation difficulties, NSSI frequency, suicidality, depression symptoms, and sleep difficulties, as well as age and global functioning. Indeed, in the original report of the trial (Bjureberg et al., 2023), other client characteristics were also not found to predict or moderate treatment outcome, including psychotropic medication use, type and frequency of other counseling, and client sexual orientation. These explorative findings are consistent with the theoretical basis of IERITA, which was designed to be a transdiagnostic treatment for NSSI among adolescents, as well as findings from a previous study of few significant predictors of treatment response to ERGT (Gratz et al., 2014). Alternatively, the absence of significant moderators could indicate constrained statistical power to detect moderation effects of smaller magnitudes. Individual moderation effects are likely to be small (Kraemer, 2013) and, thus, may require a larger sample size to detect. Additional research examining moderators and predictors of response to IERITA within larger and more diverse samples is needed before conclusions can be drawn regarding the broad utility of IERITA. Future research should also examine if the combination of individual moderators into a composite moderator (Kraemer, 2013) would create a stronger moderation effect than any single moderator tested in this study.

The negative effect of pre‐treatment parental invalidation on treatment outcome within this study is in line with findings from a previous study where adolescents (*n* = 38) received either DBT‐A or IGST (Adrian et al., 2018); they found that higher pre‐treatment parental invalidation predicted greater self‐injury after 6 months of treatment regardless of treatment condition (Adrian et al., 2018). Using a larger study sample and shorter treatment period, the present study extends previous findings by suggesting that high levels of pre‐treatment parental invalidation could predict less favorable NSSI outcomes. Although exploratory, these findings are in accordance with Linehan's biopsychosocial model (Linehan, 1993), which suggests that emotion regulation difficulties and related behaviors such as NSSI are maintained by invalidating environments. Specifically, this theory suggests that invalidation from a parent may hinder the development of adaptive emotion regulation skills and, thus, contribute to the development and maintenance of NSSI. Although results of this study suggest that high parental invalidation predicted poorer treatment response regardless of condition (i.e., the interaction term including treatment condition was not statistically significant), it is important to note that, as indicated by Figure 1, this poorer response seem to have taken the form of increased NSSI in the TAU‐only condition (vs. marginally smaller improvements in NSSI in the IERITA plus TAU condition). Future research is needed to examine if these findings replicate in other samples, and if so, the specific impact of high levels of parental invalidation on treatment outcomes among adolescents receiving TAU. Future research should also examine if and how parental invalidation may decrease during treatment and how such changes may influence adolescent response to treatment.

### Strengths and limitations

The strengths of this study include the RCT design (which allows for the examination of moderators in addition to predictors of treatment response); the use of valid and reliable measures of moderators/predictors and outcomes; the examination of a more conservative intent‐to‐treat sample; and the inclusion of weekly outcome assessments (which provides greater precision in the assessment of NSSI and may limit recall bias).

Beyond the potentially constrained statistical power as discussed above, there are some other limitations worth highlighting when interpretating the results. Our sample consisted mainly of girls and our study used the tentative criteria for NSSID (American Psychiatric Association, 2013); thus, the extent to which our findings generalize to other genders or self‐injuring adolescents without NSSID remains unclear. Furthermore, 39% of the participants were self‐referred and may not be representative of patients within child and adolescent mental health services. Still, most of the participants were clinician‐referred, and the amount of ongoing other treatment and clinical characteristics of the sample (as presented in the Methods section) suggests that this was a clinically representative sample. Finally, the presence of a moderate level of missing data during some weeks could threaten the validity of the findings, although previous inspections of patterns of data missingness (Bjureberg et al., 2023) suggest that data were likely missing at random.

## CONCLUSION

We did not find evidence of a differential treatment effect as a function of any of the examined client factors, including emotion regulation difficulties, parental invalidation, suicidality, prior NSSI, depressive symptoms, sleep difficulties, global functioning, or age. Future research should investigate moderators of IERITA in larger samples and with sufficient statistical power to detect moderation effects of smaller magnitude. As for predictors of treatment response within this trial, exploratory findings that parental invalidation had a negative impact on treatment response across both treatment conditions highlight the importance of further investigating parental invalidation in the context of NSSI treatments.

## AUTHOR CONTRIBUTIONS

**Olivia Ojala**: Conceptualization; formal analysis; investigation; methodology; writing – original draft; writing – review & editing. **Hugo Hesser**: Formal analysis; methodology; writing – original draft; writing – review & editing. **Kim L. Gratz**: Conceptualization; methodology; writing – review & editing. **Matthew T. Tull**: Conceptualization; methodology; supervision; writing – review & editing. **Erik Hedman‐Lagerlöf**: Conceptualization; methodology; supervision; writing – review & editing. **Hanna Sahlin**: Conceptualization; methodology; writing – review & editing. **Brjánn Ljótsson**: Conceptualization; methodology; supervision; writing – review & editing. **Clara Hellner**: Conceptualization; funding acquisition; investigation; methodology; project administration; supervision; writing – review & editing. **Johan Bjureberg**: Conceptualization; formal analysis; funding acquisition; investigation; methodology; project administration; supervision; writing – original draft; writing – review & editing.

## CONFLICT OF INTEREST STATEMENT

Ojala, Hesser, Hedman‐Lagerlöf, Ljótsson, and Hellner declare no competing interests. Sahlin and Bjureberg receive royalties from Natur & Kultur. Gratz receives royalties from New Harbinger Publications and Cambridge University Press. Tull receives royalties from Academic Press and New Harbinger Publications.

## ETHICAL CONSIDERATIONS

The study was approved by ethical review board in Sweden (ref no 2017/1807‐31) and was registered at Clinicaltrials.gov (Identifier NCT03353961). The adolescents and parent(s) provided oral and written informed consent (adolescents aged ≥15 provided written informed consent, whereas parents provided written informed consent on behalf of adolescents aged ≤14).

## Supporting information

Supporting Information S1

## Data Availability

Patient‐level data are not publicly available due to national (Swedish) and EU legislation but could be made available from the corresponding author upon reasonable request following approval from the Swedish Ethical Review Authority.
