# Supplementary material for: Moderators and predictors of treatment outcome following adjunctive internet‐delivered emotion regulation therapy relative to treatment as usual alone for adolescents with nonsuicidal self‐injury disorder: Randomized controlled trial
Source: JCPP Adv. 2024 May 6;4(3):e12243. doi: 10.1002/jcv2.12243 (PMC11472806; doi:10.1002/jcv2.12243)
Supplement: Supplementary file 1 — Supporting Information S1 [file JCV2-4-e12243-s001.docx]

**Supporting information**

Figure S1. Flow diagram of patient enrollment and disposition


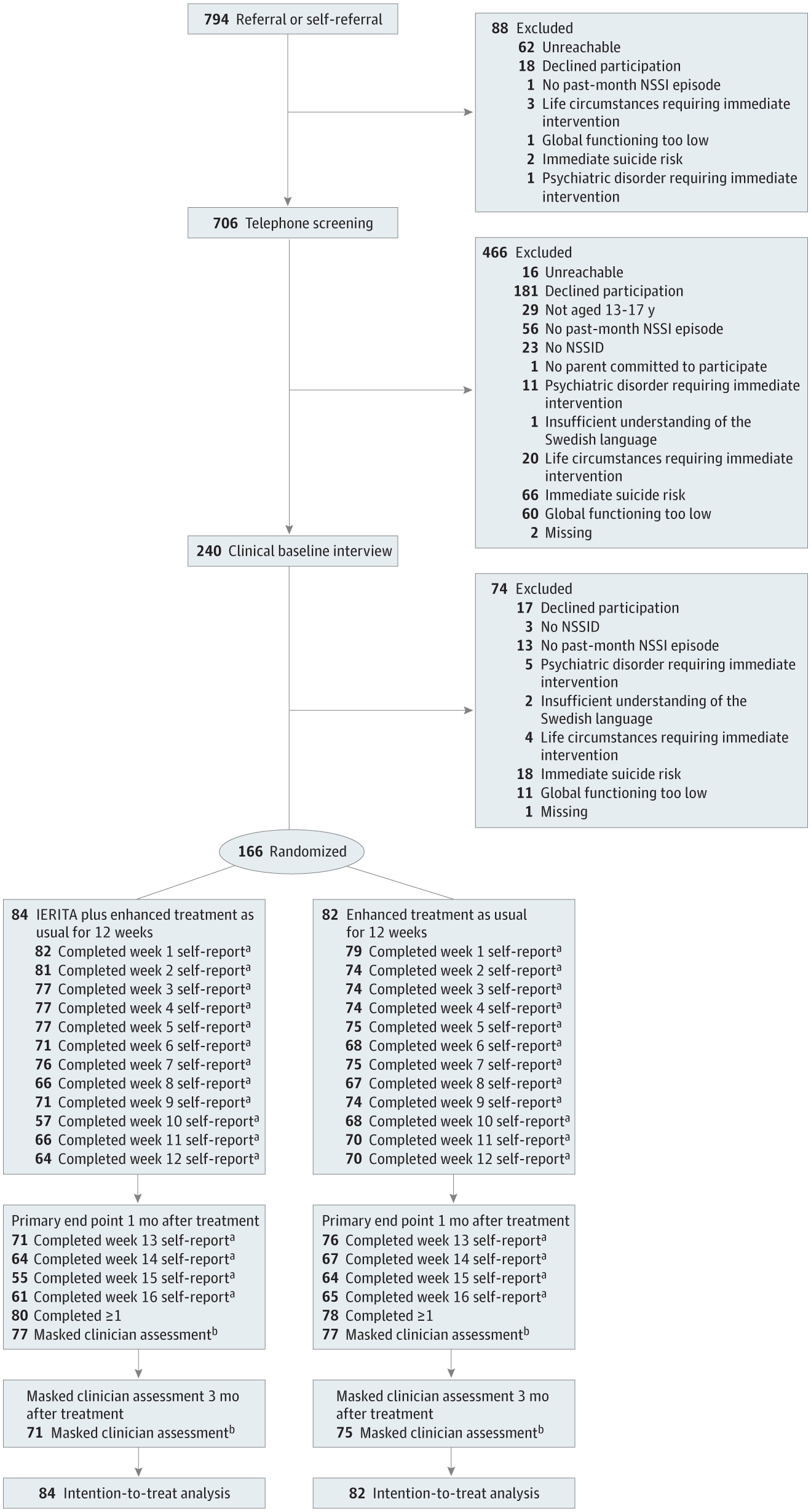


*Note.* Published in: Bjureberg J, Ojala O, Hesser H, et al. Effect of Internet-Delivered Emotion Regulation Individual Therapy for Adolescents With Nonsuicidal Self-Injury Disorder: A Randomized Clinical Trial. *JAMA Netw Open.* 2023;6(7):e2322069. doi:10.1001/jamanetworkopen.2023.22069. IERITA = internet-delivered emotion regulation individual therapy for adolescents; NSSID = nonsuicidal self-injury disorder.

^a^Self-reported Deliberate Self-Harm Inventory–Youth Version.

^b^Masked Assessor-rated Deliberate Self-Harm Inventory–Youth Version

Table S1. Pearson’s correlations for inter-correlations of moderators/predictors

|  | Age | ER difficulties | Depressive symptoms | Global functioning | Parental invalidation | Sleep difficulties | High suicidality | Prior NSSI |
| --- | --- | --- | --- | --- | --- | --- | --- | --- |
| Age | 1.00 |  |  |  |  |  |  |  |
| ER difficulties | 0.08 | 1.00 |  |  |  |  |  |  |
| Depressive symptoms | -0.06 | 0.54 | 1.00 |  |  |  |  |  |
| Global functioning | -0.05 | -0.25 | -0.28 | 1.00 |  |  |  |  |
| Parental invalidation | 0.05 | 0.04 | 0.03 | <0.01 | 1.00 |  |  |  |
| Sleep difficulties | -0.01 | 0.25 | 0.11 | -0.11 | -0.04 | 1.00 |  |  |
| High suicidality | 0.05 | 0.10 | 0.19 | -0.33 | -0.06 | 0.21 | 1.00 |  |
| Prior NSSI | -0.05 | 0.36 | 0.28 | -0.18 | <0.01 | 0.09 | 0.22 | 1.00 |

*Note.* For associations between continuous and binary variables point-biserial correlation was used. For associations between binary variables the Phi coefficient of correlation was used. ER = emotion regulation; NSSI = nonsuicidal self-injury

Table S2. Additional distributional aspects of moderators/predictors

|  | *Skewness* | *Kurtosis* |
| --- | --- | --- |
| **Moderator/predictor** |  |  |
| Age | 0.26 | -0.76 |
| Depressive symptoms | -0.21 | -0.71 |
| ER difficulties | -0.60 | -0.01 |
| Global functioning | 0.50 | -0.11 |
| Parental invalidation | 0.64 | 0.33 |
| Sleep difficulties | 0.46 | -0.22 |
| Prior past-month NSSI | 3.07 | 10.67 |
| High suicidality | 0.87 | -1.25 |

*Note.* ER = emotion regulation

Table S3. Descriptive data on the outcome of past-week frequency of nonsuicidal self-injury

|  | **IERITA+TAU (n=84)** | | | **TAU-only (n=82)** | | |
| --- | --- | --- | --- | --- | --- | --- |
|  | *No obs. (%)* | *Mean (SD)* | *Median (Q1, Q3)* | *No obs. (%)* | *Mean (SD)* | *Median (Q1, Q3)* |
| Week 0 | 84 (100) | 3.2 (3.7) | 2.0 (1.0, 5.0) | 82 (100) | 3.1 (4.0) | 2.0 (1.0, 4.0) |
| Week 1 | 82 (97.6) | 3.0 (3.9) | 2.0 (0.0, 4.0) | 79 (96.3) | 2.2 (2.4) | 2.0 (1.0, 4.0) |
| Week 2 | 81 (96.4) | 2.4 (3.3) | 1.0 (0.0, 3.0) | 74 (90.2) | 2.2 (2.6) | 1.0 (0.0, 3.0) |
| Week 3 | 77 (91.7) | 2.6 (4.0) | 1.0 (0.0, 3.0) | 74 (90.2) | 2.1 (2.5) | 2.0 (0.0, 3.0) |
| Week 4 | 77 (91.7) | 2.2 (3.7) | 1.0 (0.0, 3.0) | 74 (90.2) | 2.0 (2.1) | 1.0 (0.0, 4.0) |
| Week 5 | 77 (91.7) | 1.9 (3.1) | 1.0 (0.0, 2.0) | 75 (91.5) | 1.7 (2.5) | 2.0 (0.0, 3.0) |
| Week 6 | 71 (84.5) | 1.9 (3.1) | 1.0 (0.0, 2.0) | 68 (82.9) | 1.7 (2.1) | 1.0 (0.0, 3.0) |
| Week 7 | 76 (90.5) | 1.8 (3.5) | 0.0 (0.0, 2.0) | 75 (91.5) | 1.9 (2.8) | 1.0 (0.0, 3.0) |
| Week 8 | 66 (78.6) | 1.8 (3.7) | 0.0 (0.0, 2.0) | 67 (81.7) | 1.6 (2.2) | 1.0 (0.0, 2.0) |
| Week 9 | 71 (84.5) | 1.7 (3.1) | 0.0 (0.0, 2.0) | 74 (90.2) | 1.7 (2.3) | 1.0 (0.0, 2.0) |
| Week 10 | 57 (67.9) | 1.4 (3.2) | 0.0 (0.0, 1.0) | 68 (82.9) | 1.4 (2.3) | 1.0 (0.0, 2.0) |
| Week 11 | 66 (78.6) | 1.3 (3.5) | 0.0 (0.0, 1.0) | 70 (85.4) | 1.4 (2.4) | 0.0 (0.0, 2.0) |
| Week 12 | 64 (76.2) | 1.1 (2.5) | 0.0 (0.0, 1.0) | 70 (85.4) | 1.7 (2.9) | 0.5 (0.0, 2.0) |
| Week 13 | 71 (84.5) | 0.9 (2.3) | 0.0 (0.0, 1.0) | 76 (92.7) | 1.5 (2.4) | 0.0 (0.0, 3.0) |
| Week 14 | 64 (76.2) | 0.8 (1.6) | 0.0 (0.0, 1.0) | 67 (81.7) | 1.5 (2.6) | 0.0 (0.0, 2.0) |
| Week 15 | 55 (65.5) | 0.8 (2.4) | 0.0 (0.0, 1.0) | 64 (78.0) | 1.5 (2.7) | 0.0 (0.0, 2.0) |
| Week 16 | 61 (72.6) | 0.9 (2.3) | 0.0 (0.0, 1.0) | 65 (79.3) | 1.4 (2.7) | 0.0 (0.0, 2.0) |

*Note.* Data in this table has also been presented in: Bjureberg J, Ojala O, Hesser H, et al. Effect of Internet-Delivered Emotion Regulation Individual Therapy for Adolescents With Nonsuicidal Self-Injury Disorder: A Randomized Clinical Trial. *JAMA Netw Open.* 2023;6(7):e2322069. doi:10.1001/jamanetworkopen.2023.22069. IERITA = Internet-delivered Emotion Regulation Individual Therapy; ER = Emotion regulation; TAU = Treatment as Usual; SD = standard deviation; Q = quartile

Table S4. Key parameter estimates obtained from generalized linear mixed models examining change in self-rated NSSI episodes from pre-treatment to post-treatment

|  | **Estimate (b)** | **SE** | **z-value** | **p-value** |
| --- | --- | --- | --- | --- |
| **Age** |  |  |  |  |
| Intercept | 0.884 | 0.125 | 7.059 | 0.000 |
| Treatment | 0.191 | 0.199 | 0.961 | 0.337 |
| Time | 0.006 | 0.018 | 0.337 | 0.736 |
| Age | -0.062 | 0.119 | -0.521 | 0.602 |
| Treatment x Time | -0.077 | 0.022 | -3.544 | 0.000 |
| Age x Treatment | 0.038 | 0.153 | 0.252 | 0.801 |
| Age x Time | 0.018 | 0.011 | 1.666 | 0.096 |
| Age x Time x Treatment | -0.018 | 0.017 | -1.064 | 0.287 |
| **High Suicidality** |  |  |  |  |
| Intercept | 0.694 | 0.153 | 4.531 | 0.000 |
| Treatment | 0.315 | 0.244 | 1.291 | 0.197 |
| Time | 0.010 | 0.021 | 0.478 | 0.633 |
| High Suicidality | 0.559 | 0.254 | 2.201 | 0.028 |
| Treatment x Time | -0.084 | 0.025 | -3.380 | 0.001 |
| High Suicidality x Treatment | -0.404 | 0.388 | -1.042 | 0.297 |
| High Suicidality x Time | -0.004 | 0.029 | -0.145 | 0.885 |
| High Suicidality x Time x Treatment | 0.015 | 0.046 | 0.329 | 0.742 |
| **Global Functioning** |  |  |  |  |
| Intercept | 0.841 | 0.134 | 6.286 | 0.000 |
| Treatment | 0.171 | 0.175 | 0.973 | 0.331 |
| Time | 0.005 | 0.018 | 0.271 | 0.786 |
| Global Functioning | -0.072 | 0.019 | -3.818 | 0.000 |
| Treatment x Time | -0.082 | 0.022 | -3.676 | 0.000 |
| Global Functioning x Treatment | 0.038 | 0.034 | 1.104 | 0.270 |
| Global Functioning x Time | -0.001 | 0.001 | -0.712 | 0.477 |
| Global Functioning x Time x Treatment | -0.004 | 0.004 | -0.974 | 0.330 |
| **Parental invalidation** |  |  |  |  |
| Intercept | 0.917 | 0.126 | 7.272 | 0.000 |
| Treatment | 0.183 | 0.198 | 0.926 | 0.354 |
| Time | 0.003 | 0.016 | 0.200 | 0.841 |
| Parental invalidation | 0.154 | 0.139 | 1.113 | 0.266 |
| Treatment x Time | -0.080 | 0.023 | -3.544 | 0.000 |
| Parental invalidation x Treatment | -0.288 | 0.182 | -1.579 | 0.114 |
| Parental invalidation x Time | 0.027 | 0.011 | 2.455 | 0.014 |
| Parental invalidation x Time x Treatment | -0.019 | 0.017 | -1.068 | 0.285 |
| **Depressive symptoms** |  |  |  |  |
| Intercept | 0.757 | 0.136 | 5.548 | 0.000 |

Table S4. Key parameter estimates obtained from generalized linear mixed models examining change in self-rated NSSI episodes from pre-treatment to post-treatment (continued)

|  | **Estimate (b)** | **SE** | **z-value** | **p-value** |
| --- | --- | --- | --- | --- |
| Treatment | 0.238 | 0.167 | 1.426 | 0.154 |
| Time | 0.004 | 0.019 | 0.203 | 0.839 |
| Depressive symptoms | 0.118 | 0.027 | 4.343 | 0.000 |
| Treatment x Time | -0.081 | 0.022 | -3.628 | 0.000 |
| Depressive symptoms x Treatment | -0.039 | 0.036 | -1.098 | 0.272 |
| Depressive symptoms x Time | -0.001 | 0.003 | -0.246 | 0.806 |
| Depressive symptoms x Time x Treatment | 0.005 | 0.005 | 1.181 | 0.238 |
| **ER difficulties** |  |  |  |  |
| Intercept | 0.704 | 0.123 | 5.704 | 0.000 |
| Treatment | 0.291 | 0.173 | 1.684 | 0.092 |
| Time | -0.013 | 0.015 | -0.894 | 0.371 |
| ER difficulties | 0.034 | 0.007 | 5.103 | 0.000 |
| Treatment x Time | -0.082 | 0.020 | -4.062 | 0.000 |
| ER difficulties x Treatment | -0.013 | 0.009 | -1.427 | 0.153 |
| ER difficulties x Time | 0.001 | 0.001 | 1.256 | 0.209 |
| ER difficulties x Time x Treatment | 0.002 | 0.001 | 1.600 | 0.110 |
| **Sleep problems** |  |  |  |  |
| Intercept | 0.877 | 0.116 | 7.561 | 0.000 |
| Treatment | 0.193 | 0.183 | 1.051 | 0.293 |
| Time | 0.007 | 0.018 | 0.403 | 0.687 |
| Sleep problems | 0.011 | 0.021 | 0.501 | 0.617 |
| Treatment x Time | -0.078 | 0.020 | -3.958 | 0.000 |
| Sleep problems x Treatment | 0.025 | 0.033 | 0.782 | 0.434 |
| Sleep problems x Time | 0.001 | 0.003 | 0.430 | 0.667 |
| Sleep problems x Time x Treatment | -0.003 | 0.004 | -0.713 | 0.476 |
| **Prior NSSI** |  |  |  |  |
| Intercept | -0.127 | 0.175 | -0.725 | 0.469 |
| Treatment | 0.281 | 0.232 | 1.214 | 0.225 |
| Time | 0.021 | 0.019 | 1.131 | 0.258 |
| Prior NSSI | 1.505 | 0.211 | 7.131 | 0.000 |
| Treatment x Time | -0.097 | 0.032 | -3.095 | 0.002 |
| Prior NSSI x Treatment | -0.121 | 0.279 | -0.435 | 0.664 |
| Prior NSSI x Time | -0.021 | 0.027 | -0.785 | 0.433 |
| Prior NSSI x Time x Treatment | 0.036 | 0.042 | 0.839 | 0.401 |

*Note.* Marginal coefficients with robust standard errors are presented. Reference group for treatment is treatment as usual (control condition). Reference group for high suicidality is moderate or low suicidality. Reference group for prior NSSI is <8 episodes. ER = emotion regulation; NSSI = nonsuicidal self-injury; SE = standard error

Table S5. Key parameter estimates obtained from generalized linear mixed models examining change in self-rated NSSI episodes from pre-treatment to post-treatment as a function of treatment condition: sensitivity analysis of age groups

|  | **Estimate (b)** | **SE** | **z-value** | **p-value** |
| --- | --- | --- | --- | --- |
| **Age group** |  |  |  |  |
| Moderator | -0.025 | 0.036 | -0.700 | 0.484 |
| Predictor | 0.029 | 0.025 | 1.144 | 0.253 |

*Note.* Moderator corresponds to the interactions of client factor x time x treatment. Predictor corresponds to the interaction of client factor x time. Marginal coefficients with robust standard errors are presented. Reference group for age group is 13 to 14 years old. SE = standard error
